# Supplementary material for: RNAseq Analysis of FABP4 Knockout Mouse Hippocampal Transcriptome Suggests a Role for WNT/β-Catenin in Preventing Obesity-Induced Cognitive Impairment
Source: Int J Mol Sci. 2023 Feb 8;24(4):3381. doi: 10.3390/ijms24043381 (PMC9961923; doi:10.3390/ijms24043381)
Supplement: Supplementary file 1 [file ijms-24-03381-s001.zip › ijms-2147764-supplementary.pdf]

Supplementary Materials

S1. Results

S1.1. Differentially Expressed Pathways in HFD-Fed AKO Compared to HFD-fed WT Mice

Using Reactome molecular pathway analysis, we determined the differentially expressed pathways in HFD-fed AKO mice compared to HFD-fed WT mice (FDR ≤ 0.01). There were 39 pathways in total. 9 of these pathways were unrelated to ribosomal protein subunits and were examined further for transcript function (Supplemental Table S1). These 9 differentially expressed pathways were related to metabolism. Transcripts associated with the electron transport chain [1–8] and citric acid cycle [9–12] were downregulated and upregulated, respectively. Transcripts associated with Golgi transport were upregulated [13–45]. Transcripts relating to mRNA polyadenylation were upregulated [46–51]. Transcripts relating to Wnt/β-Catenin signaling were upregulated [52–57].

Supplemental Table S1. Differentially Expressed Pathways in HFD-fed AKO Compared to HFD-fed WT Mice. Pathways with FDR ≤ 0.01 are displayed. Pathway name, stable identifier, log fold change (log2), and false discovery rate for each pathway are displayed. Within each pathway, gene ID, Ensembl ID, gene description, log fold change (log2), log10 adjusted *p*-value, and relevant known function are displayed.

Reactome Pathway	Gene Symbol	Ensembl ID	Gene Description	Log FC	*p*adj	Known Function
Formation of ATP by chemiosmotic coupling (R-HSA-163210) LFC: -0.13 FDR: 2.40×10^{-4}	*Atp5j2*<br>*Atp5h*	ENSMUST00000161741<br>ENSMUST00000043931	ATP synthase, H+ transporting, mitochondrial F0 complex, subunit F2<br>ATP synthase, H+ transporting, mitochondrial F0 complex, subunit D	-0.27 -<br>0.26	1.82×10^{-2}<br>3.98×10^{-2}	Inhibition is neuroprotective in aging and AD [58]
Cristae formation (R-HSA-8949613) LFC: -0.09 FDR: 6.07×10^{-3}	*Atp5j2*<br>*Atp5h*	ENSMUST00000161741<br>ENSMUST00000043931	ATP synthase, H+ transporting, mitochondrial F0 complex, subunit F2<br>ATP synthase, H+ transporting, mitochondrial F0 complex, subunit D	-0.27 -<br>0.26	1.82×10^{-2}<br>3.98×10^{-2}	Inhibition is neuroprotective in aging and AD [58]
	Hspa9	ENSMUST00000025217	Heat shock protein 9	0.28	1.69×10^{-2}	Oxidative stress sensor [59], Downregulated in AD brain [60]
	Samm50	ENSMUST00000023071	SAMM50 sorting and assembly machinery component	0.37	1.12×10^{-2}	Negative regulator of mitochondrial fragmentation [61]
Complex I biogenesis (R-HSA-6799198) LFC: -0.04 FDR: 3.00×10^{-4}	*Ndufb2*<br>*Ndufb4*<br>*Ndufb11*<br>*Ndufs8*<br>*Ndufa4*<br>*Ndufb9*<br>*Ndufa2*	ENSMUST00000119379<br>ENSMUST00000023514<br>ENSMUST00000116621<br>ENSMUST00000237341<br>ENSMUST00000204978<br>ENSMUST00000022980<br>ENSMUST00000014438	NADH:ubiquinone oxidoreductase subunit B2<br>NADH:ubiquinone oxidoreductase subunit B4<br>NADH:ubiquinone oxidoreductase subunit B11<br>NADH:ubiquinone oxidoreductase core subunit S8<br>Ndufa4, mitochondrial complex associated<br>NADH:ubiquinone oxidoreductase subunit B9<br>NADH:ubiquinone oxidoreductase subunit A2	-0.32 -<br>0.29 -<br>0.28 -<br>0.27 -<br>0.25 -<br>0.23 -<br>0.20	2.39×10^{-2}<br>2.89×10^{-2}<br>3.32×10^{-2}<br>4.63×10^{-2}<br>4.69×10^{-2}<br>4.16×10^{-2}<br>4.83×10^{-2}	Partial inhibition of Complex I leads to reduced oxidative stress and increased long-term potentiation in AD mice [1]

	mt-Nd3	ENSMUST00000082411	Mitochondrially encoded NADH dehydrogenase 3	-0.23	4.06×10^{-2}	Partial inhibition of Complex I leads to reduced oxidative stress and increased long-term potentiation in AD mice [1]
	Acad9	ENSMUST00000011492	Acyl-Coenzyme A dehydrogenase family, member 9	0.29	2.59×10^{-2}	Role in fatty acid oxidation, downregulation associated with neurological disease [2]
	Timmdc1	ENSMUST00000002925	Translocase of inner mitochondrial membrane domain containing 1	0.36	9.17×10^{-3}	Loss is associated with axonal neuropathy and cognitive decline [3]
Respiratory electron transport, ATP synthesis by chemiosmotic coupling, and heat production by uncoupling proteins (R-HSA-163200) LFC: -0.04 FDR: 8.45×10^{-7}	*Ndufb2*<br>*Ndufb4*<br>*Ndufb11*<br>*Ndufs8*<br>*Ndufa4*<br>*Ndufb9*<br>*Ndufa2*	ENSMUST00000119379<br>ENSMUST00000023514<br>ENSMUST00000116621<br>ENSMUST00000237341<br>ENSMUST00000204978<br>ENSMUST00000022980<br>ENSMUST00000014438	NADH:ubiquinone oxidoreductase subunit B2<br>NADH:ubiquinone oxidoreductase subunit B4<br>NADH:ubiquinone oxidoreductase subunit B11<br>NADH:ubiquinone oxidoreductase core subunit S8<br>Ndufa4, mitochondrial complex associated<br>NADH:ubiquinone oxidoreductase subunit B9<br>NADH:ubiquinone oxidoreductase subunit A2	-0.32 -<br>0.29 -<br>0.28 -<br>0.27 -<br>0.25 -<br>0.23 -<br>0.20	2.39×10^{-2}<br>2.89×10^{-2}<br>3.32×10^{-2}<br>4.63×10^{-2}<br>4.69×10^{-2}<br>4.16×10^{-2}<br>4.83×10^{-2}	Partial inhibition of Complex I leads to reduced oxidative stress and increased long-term potentiation in AD mice [1]
	Cox7b	ENSMUST00000033582	Cytochrome c oxidase subunit 7B	-0.31	1.13×10^{-2}	Loss leads to decrease in oxidative stress and amyloid formation in AD mice [4]
	Uqcrh	ENSMUST00000078676	Ubiquinol-cytochrome c reductase hinge protein	-0.29	3.04×10^{-2}	Positive regulator of apoptosis via cytochrome c release [5]
	Atp5j2<br>*Atp5h*	ENSMUST00000161741<br>ENSMUST00000043931	ATP synthase, H+ transporting, mitochondrial F0 complex, subunit F2<br>ATP synthase, H+ transporting, mitochondrial F0 complex, subunit D	-0.27 -<br>0.26	1.82×10^{-2}<br>3.98×10^{-2}	Inhibition is neuroprotective in aging and AD [58]
	mt-Nd3	ENSMUST00000082411	Mitochondrially encoded NADH dehydrogenase 3	-0.23	4.06×10^{-2}	Partial inhibition of Complex I leads to reduced oxidative stress and increased long-term potentiation in AD mice [1]
	mt-Co3	ENSMUST00000082409	Mitochondrially encoded cytochrome c oxidase III	-0.21	3.87×10^{-2}	Loss leads to decrease in oxidative stress and amyloid formation in AD mice [4]
	mt-Cytb	ENSMUST00000082421	Mitochondrially encoded cytochrome b	0.21	4.53×10^{-2}	Decreased in AD brain [6]
	Acad9	ENSMUST00000011492	Acyl-Coenzyme A dehydrogenase family, member 9	0.29	2.59×10^{-2}	Role in fatty acid oxidation, downregulation associated with neurological disease [2]
	Sdha	ENSMUST00000022062	Succinate dehydrogenase complex, subunit A, flavoprotein (Fp)	0.29	1.02×10^{-2}	Downregulation is associated with oxidative stress and insulin resistance [7]
	Timmdc1	ENSMUST00000002925	Translocase of inner mitochondrial membrane domain containing 1	0.36	9.17×10^{-3}	Loss is associated with axonal neuropathy and cognitive decline [3]
	Taco1	ENSMUST00000002048	Translational activator of mitochondrially encoded cytochrome c oxidase I	0.40	3.68×10^{-2}	Loss associated with motor disfunction and mitochondrial disease in mice [8]

Respiratory electron transport (R-HSA-611105)<br>LFC: -0.03<br>FDR: 2.92 × 10⁻⁵	*Ndufb2*<br>*Ndufb4*<br>*Ndufb11*<br>*Ndufs8*<br>*Ndufa4*<br>*Ndufb9*<br>*Ndufa2*	ENSMUST00000119379<br>ENSMUST00000023514<br>ENSMUST00000116621<br>ENSMUST00000237341<br>ENSMUST00000204978<br>ENSMUST00000022980<br>ENSMUST00000014438	NADH:ubiquinone oxidoreductase subunit B2<br>NADH:ubiquinone oxidoreductase subunit B4<br>NADH:ubiquinone oxidoreductase subunit B11<br>NADH:ubiquinone oxidoreductase core subunit S8<br>Ndufa4, mitochondrial complex associated<br>NADH:ubiquinone oxidoreductase subunit B9<br>NADH:ubiquinone oxidoreductase subunit A2	-0.32 -<br>0.29 -<br>0.28 -<br>0.27 -<br>0.25 -<br>0.23 -<br>0.20	2.39×10^{-2}<br>2.89×10^{-2}<br>3.32×10^{-2}<br>4.63×10^{-2}<br>4.69×10^{-2}<br>4.16×10^{-2}<br>4.83×10^{-2}	Partial inhibition of Complex I leads to reduced oxidative stress and increased long-term potentiation in AD mice [1]
	Cox7b	ENSMUST00000033582	Cytochrome c oxidase subunit 7B	-0.31	1.13×10^{-2}	Loss leads to decrease in oxidative stress and amyloid formation in AD mice [4]
	Uqcrh	ENSMUST00000078676	Ubiquinol-cytochrome c reductase hinge protein	-0.29	3.04×10^{-2}	Positive regulator of apoptosis via cytochrome c release [5]
	mt-Nd3	ENSMUST00000082411	Mitochondrially encoded NADH dehydrogenase 3	-0.23	4.06×10^{-2}	Partial inhibition of Complex I leads to reduced oxidative stress and increased long-term potentiation in AD mice [1]
	mt-Co3	ENSMUST00000082409	Mitochondrially encoded cytochrome c oxidase III	-0.21	3.87×10^{-2}	Loss leads to decrease in oxidative stress and amyloid formation in AD mice [4]
	mt-Cytb	ENSMUST00000082421	Mitochondrially encoded cytochrome b	0.21	4.53×10^{-2}	Decreased in AD brain [6]
	Acad9	ENSMUST00000011492	Acyl-Coenzyme A dehydrogenase family, member 9	0.29	2.59×10^{-2}	Role in fatty acid oxidation, downregulation associated with neurological disease [2]
	Sdha	ENSMUST00000022062	Succinate dehydrogenase complex, subunit A, flavoprotein (Fp)	0.29	1.02×10^{-2}	Downregulation is associated with oxidative stress and insulin resistance [7]
	Timmdc1	ENSMUST00000002925	Translocase of inner mitochondrial membrane domain containing 1	0.36	9.17×10^{-3}	Loss is associated with axonal neuropathy and cognitive decline [3]
	Taco1	ENSMUST00000002048	Translational activator of mitochondrially encoded cytochrome c oxidase I	0.40	3.68×10^{-2}	Loss associated with motor disfunction and mitochondrial disease in mice [8]
The citric acid (TCA) cycle and respiratory electron transport (R-HSA-1428517)<br>LFC: 0.03<br>FDR: 2.71 × 10⁻³	*Ndufb2*<br>*Ndufb4*<br>*Ndufb11*<br>*Ndufs8*<br>*Ndufa4*<br>*Ndufb9*<br>*Ndufa2*	ENSMUST00000119379<br>ENSMUST00000023514<br>ENSMUST00000116621<br>ENSMUST00000237341<br>ENSMUST00000204978<br>ENSMUST00000022980<br>ENSMUST00000014438	NADH:ubiquinone oxidoreductase subunit B2<br>NADH:ubiquinone oxidoreductase subunit B4<br>NADH:ubiquinone oxidoreductase subunit B11<br>NADH:ubiquinone oxidoreductase core subunit S8<br>Ndufa4, mitochondrial complex associated<br>NADH:ubiquinone oxidoreductase subunit B9<br>NADH:ubiquinone oxidoreductase subunit A2	-0.32 -<br>0.29 -<br>0.28 -<br>0.27 -<br>0.25 -<br>0.23 -<br>0.20	2.39×10^{-2}<br>2.89×10^{-2}<br>3.32×10^{-2}<br>4.63×10^{-2}<br>4.69×10^{-2}<br>4.16×10^{-2}<br>4.83×10^{-2}	Partial inhibition of Complex I leads to reduced oxidative stress and increased long-term potentiation in AD mice [1]
	Cox7b	ENSMUST00000033582	Cytochrome c oxidase subunit 7B	-0.31	1.13×10^{-2}	Loss leads to decrease in oxidative stress and amyloid formation in AD mice [4]

	Uqcrh	ENSMUST00000078676	Ubiquinol-cytochrome c reductase hinge protein	-0.29	3.04×10^{-2}	Positive regulator of apoptosis via cytochrome c release [5]
	Atp5j2 *Atp5h*	ENSMUST00000161741 ENSMUST00000043931	ATP synthase, H+ transporting, mitochondrial F0 complex, subunit F2 ATP synthase, H+ transporting, mitochondrial F0 complex, subunit D	-0.27 - 0.26	1.82×10^{-2} 3.98×10^{-2}	Inhibition is neuroprotective in aging and AD [58]
	mt-Nd3	ENSMUST00000082411	Mitochondrially encoded NADH dehydrogenase 3	-0.23	4.06×10^{-2}	Partial inhibition of Complex I leads to reduced oxidative stress and increased long-term potentiation in AD mice [1]
	mt-Co3	ENSMUST00000082409	Mitochondrially encoded cytochrome c oxidase III	-0.21	3.87×10^{-2}	Loss leads to decrease in oxidative stress and amyloid formation in AD mice [4]
	mt-Cytb	ENSMUST00000082421	Mitochondrially encoded cytochrome b	0.21	4.53×10^{-2}	Decreased in AD brain [6]
	Acad9	ENSMUST00000011492	Acyl-Coenzyme A dehydrogenase family, member 9	0.29	2.59×10^{-2}	Role in fatty acid oxidation, downregulation associated with neurological disease [2]
	Aco2	ENSMUST00000023116	Aconitase 2, mitochondrial	0.29	1.42×10^{-2}	Suppressed by nitric oxide (Palmieri et al., 2020), Decreased in AD patient lymphocytes [9]
	Sdha	ENSMUST00000022062	Succinate dehydrogenase complex, subunit A, flavoprotein (Fp)	0.29	1.02×10^{-2}	Downregulation is associated with oxidative stress and insulin resistance [7]
	Ogdh	ENSMUST00000003461	Oxoglutarate (alpha-ketoglutarate) dehydrogenase (lipoamide)	0.30	3.73×10^{-2}	Inhibited by lipopolysaccharide and IFN-γ stimulation in macrophages [10]
	Cs	ENSMUST00000005826	Citrate synthase	0.32	1.53×10^{-2}	Downregulated in AD patient platelets [11]
	Pdk2	ENSMUST00000038431	Pyruvate dehydrogenase kinase, isoenzyme 2	0.35	4.52×10^{-2}	mRNA levels modulated during aging in brain [12]
	Timmdc1	ENSMUST00000002925	Translocase of inner mitochondrial membrane domain containing 1	0.36	9.17×10^{-3}	Loss is associated with axonal neuropathy and cognitive decline [3]
	Taco1	ENSMUST00000002048	Translational activator of mitochondrially encoded cytochrome c oxidase I	0.40	3.68×10^{-2}	Loss associated with motor disfunction and mitochondrial disease in mice [8]
Transport to the Golgi and subsequent modification (R-HSA-948021) LFC: 0.27 FDR: 5.71×10^{-3}	*Arcn1*	ENSMUST00000034607	Archain 1	0.24	2.71×10^{-2}	Loss is associated with increased ER stress [13]
	Dctn4 Dctn5 Dctn1	ENSMUST00000025505 ENSMUST00000033156 ENSMUST00000113919	Dynactin 4 Dynactin 5 Dynactin 1	0.25 0.30 0.56	4.42×10^{-2} 2.38×10^{-2} 2.10×10^{-2}	Positive regulators of retrograde axonal transport, decreased activity associated with neurodegeneration [14]
	Ykt6	ENSMUST00000002818	YKT6 v-SNARE homolog (S. cerevisiae)	0.26	1.97×10^{-2}	Negative regulator of α-synuclein, downregulated in PD brain [15]
	Kdelr2	ENSMUST00000110731	KDEL (Lys-Asp-Glu-Leu) endoplasmic reticulum protein retention receptor 2	0.27	4.30×10^{-2}	Regulator of ER proteome homeostasis in response to ER stress [16]

	Man1a2<br>*Man2a2*	ENSMUST00000008907<br>ENSMUST00000098346	Mannosidase, alpha, class 1A, member 2<br>Mannosidase 2, alpha 2	0.28<br>0.65	3.16×10^{-2}<br>1.70×10^{-2}	Regulators of N-glycosylation [17]
	Copz1<br>*Copg1*	ENSMUST00000100162<br>ENSMUST00000113607	Coatomer protein complex, subunit zeta 1<br>Coatomer protein complex, subunit gamma 1	0.30<br>0.46	1.59×10^{-2}<br>4.97×10^{-3}	Positive regulators of neurite outgrowth [18]
	Tbc1d20	ENSMUST00000028963	TBC1 domain family, member 20	0.32	4.24×10^{-2}	Downregulation leads to disrupted neuronal autophagic flux and motor disfunction [19]
	Cog4<br>*Cog1*	ENSMUST00000034203<br>ENSMUST00000018805	Component of oligomeric golgi complex 4<br>Component of oligomeric golgi complex 1	0.34<br>0.44	1.62×10^{-2}<br>8.60×10^{-3}	Regulator of Golgi vesicle trafficking, defects are associated with neurological disorders [20]
	Gosr2 Gosr1	ENSMUST00000021329<br>ENSMUST00000010536	Golgi SNAP receptor complex member 2<br>Golgi SNAP receptor complex member 1	0.35<br>0.38	9.17×10^{-3}<br>6.24×10^{-3}	Regulator of ER to Golgi trafficking, dysregulation associated with neurological disorders [21]
	Dynll2<br>*Dync1li1*<br>*Dync1h1*	ENSMUST00000020775<br>ENSMUST00000047404<br>ENSMUST00000018851	Dynein light chain LC8-type 2<br>Dynein cytoplasmic 1 light intermediate chain 1<br>Dynein cytoplasmic 1 heavy chain 1	0.36<br>0.38<br>0.50	5.25×10^{-3}<br>7.11×10^{-3}<br>1.28×10^{-2}	Positive regulators of retrograde axonal transport [22], Downregulated in AD hippocampus [23]
	Sec22b<br>*Sec24b*<br>*Sec31a*<br>*Sec24c*<br>*Sec22c*	ENSMUST00000029476<br>ENSMUST00000001079<br>ENSMUST00000094578<br>ENSMUST00000048657<br>ENSMUST00000078547	SEC22 homolog B, vesicle trafficking protein<br>Sec24 related gene family, member B (S. cerevisiae)<br>Sec31 homolog A (S. cerevisiae)<br>Sec24 related gene family, member C (S. cerevisiae)<br>SEC22 homolog C, vesicle trafficking protein	0.36<br>0.40<br>0.46<br>0.49<br>0.82	3.74×10^{-2}<br>9.12×10^{-3}<br>7.12×10^{-3}<br>1.35×10^{-2}<br>3.28×10^{-3}	Regulators of vesicle trafficking, downregulated in aging and AD brains [24], Loss leads to neuronal apoptotic cell death [25]
	Tmed9	ENSMUST00000109905	Transmembrane p24 trafficking protein 9	0.38	1.50×10^{-2}	Positive regulator of autophagosome biogenesis [26]
	Napa	ENSMUST00000006181	N-ethylmaleimide sensitive fusion protein attachment protein alpha	0.41	2.59×10^{-2}	Loss leads to impairment of Golgi to ER trafficking and apoptosis [27]
	Actr1a	ENSMUST00000040270	ARP1 actin-related protein 1A, centractin alpha	0.41	4.56×10^{-3}	Positive regulator of retrograde axonal transport [28]
	Trappc5<br>*Trappc9*	ENSMUST00000044857<br>ENSMUST00000089770	Trafficking protein particle complex 5<br>Trafficking protein particle complex 9	0.42<br>0.43	6.44×10^{-3}<br>3.92×10^{-2}	Loss leads to decreased social memory and obesity in mice [29]
	Tubb6<br>*Tuba4a*	ENSMUST00000001513<br>ENSMUST00000186213	Tubulin, beta 6 class V<br>Tubulin, alpha 4A	0.45<br>0.52	4.40×10^{-2}<br>8.60×10^{-3}	Downregulation leads to motor axon defects [30]
	Tmem115	ENSMUST00000010189	Transmembrane protein 115	0.45	7.28×10^{-3}	Regulator of Golgi to ER retrograde transport [31]
	Arfgap1	ENSMUST00000029092	ADP-ribosylation factor GTPase activating protein 1	0.46	1.28×10^{-2}	Regulator of targeting of GABA transporter 1 in axon terminals [32]

	Mgat4b *Mgat5*	ENSMUST00000041725 ENSMUST00000038361	Mannoside acetylglucosaminyltransferase 4, isoenzyme B Mannoside acetylglucosaminyltransferase 5	0.47 0.70	1.50×10^{-2} 3.81×10^{-3}	Negative regulators of spontaneous inflammatory demyelination and neurodegeneration [33]
	B4galt3 *B4galt6* *B4galt5*	ENSMUST00000064272 ENSMUST00000070080 ENSMUST00000109221	UDP-Gal:betaGlcNAc beta 1,4-galactosyltransferase, polypeptide 3 UDP-Gal:betaGlcNAc beta 1,4-galactosyltransferase, polypeptide 6 UDP-Gal:betaGlcNAc beta 1,4-galactosyltransferase, polypeptide 5	0.48 0.57 0.62	3.60×10^{-2} 4.56×10^{-3} 3.27×10^{-3}	Regulators of inflammatory cytokine response in LPS-treated microglia [34]
	Uso1	ENSMUST00000031355	USO1 vesicle docking factor	0.50	9.05×10^{-3}	Positive regulator of ER to Golgi transport [35]
	St8sia3	ENSMUST00000025477	ST8 alpha-N-acetyl-neuraminide alpha-2,8-sialyltransferase 3	0.52	5.84×10^{-3}	Regulator of dopamine D_1R and D_2R receptors, downregulated in neurodegenerative disease [36]
	Cnih3	ENSMUST00000027795	Cornichon family AMPA receptor auxiliary protein 3	0.54	2.00×10^{-2}	Positive regulator of hippocampal AMPAR synaptic transmission [37]
	Arf3	ENSMUST00000053183	ADP-ribosylation factor 3	0.56	7.12×10^{-2}	Paralog ARF6 mediates BACE1 and APP processing [38]
	Ppp6r1	ENSMUST00000064099	Protein phosphatase 6, regulatory subunit 1	0.56	4.91×10^{-3}	Negative regulator of NF-κB signaling [39]
	Nsf	ENSMUST00000103075	N-ethylmaleimide sensitive fusion protein	0.59	2.66×10^{-2}	Positive regulator of synaptic AMPA receptor function [40]
	Sptbn2 *Sptbn1*	ENSMUST00000008991 ENSMUST00000011877	Spectrin beta, non-erythrocytic 2 Spectrin beta, non-erythrocytic 1	0.62 0.85	7.88×10^{-3} 4.89×10^{-3}	Loss leads to decrease in dendritic spine density and neurodegeneration [41]
	Csnk1d	ENSMUST00000070575	Casein kinase 1, delta	0.65	5.83×10^{-3}	Positive regulator of Wnt/β-catenin signaling [42]
	Lman2 *Lman2l*	ENSMUST00000021940 ENSMUST00000125304	Lectin, mannose-binding 2 Lectin, mannose-binding 2-like	0.69 0.86	5.30×10^{-3} 5.07×10^{-3}	Regulator of glycoprotein transport and sorting, dysregulation associated with neurodegenerative disease [43]
	Gbf1	ENSMUST00000026254	Golgi-specific brefeldin A-resistance factor 1	0.69	7.23×10^{-3}	Dysregulation associated with Golgi fragmentation [44]
	Fut8	ENSMUST00000062804	Fucosyltransferase 8	0.84	3.62×10^{-3}	Loss leads to increased microgliosis under LPS stimulation [45]
Processing of Intronless Pre-mRNAs (R-HSA-77595) LFC: 0.29 FDR: 7.79×10^{-3}	*Cstf2* *Cstf1 Cstf2t*	ENSMUST00000113286 ENSMUST00000116375 ENSMUST00000066039	Cleavage stimulation factor, 3' pre-RNA subunit 2 Cleavage stimulation factor, 3' pre-RNA, subunit Cleavage stimulation factor, 3' pre-RNA subunit 2, tau	-0.70 0.33 0.34	3.86×10^{-2} 1.41×10^{-2} 1.29×10^{-2}	Positive regulators of 3′ cleavage and polyadenylation [46]
	Nudt21	ENSMUST00000034204	Nudix (nucleoside diphosphate linked moiety X)-type motif 21	0.23	2.59×10^{-2}	Loss leads to dysregulated hippocampal alternative polyadenylation and learning deficits [47]

	Ncbp1 *Ncbp2*	ENSMUST00000030014 ENSMUST00000023460	Nuclear cap binding protein subunit 1 Nuclear cap binding protein subunit 2	0.25 0.28	3.87×10^{-2} 1.48×10^{-2}	Positive regulator of mRNA export from the nucleus [48]
	Cpsf7 Cpsf2	ENSMUST00000038379 ENSMUST00000047357	Cleavage and polyadenylation specific factor 7 Cleavage and polyadenylation specific factor 2	0.28 0.34	2.41×10^{-2} 1.26×10^{-2}	Positive regulators of 3′ cleavage and polyadenylation [46]
	Wdr33	ENSMUST00000025264	WD repeat domain 33	0.29	2.75×10^{-2}	Positive regulator of polyadenylation signal recognition [49]
	Papola	ENSMUST00000109901	Poly (A) polymerase alpha	0.39	3.72×10^{-2}	Positive regulator of polyadenylation [46]
	Sympk	ENSMUST00000023882	Symplekin	0.48	7.15×10^{-3}	Positive regulator of polyadenylation [50], Downregulated in AD brain [51]
Disassembly of the destruction complex and recruitment of AXIN to the membrane (R-HSA-4641262) LFC: 0.38 FDR: 7.21×10^{-3}	*Ppp2cb* *Ppp2r5a* *Ppp2r5e* *Ppp2r1a*	ENSMUST00000009774 ENSMUST00000067976 ENSMUST00000021447 ENSMUST00000007708	Protein phosphatase 2 (formerly 2A), catalytic subunit, beta isoform Protein phosphatase 2, regulatory subunit B', alpha Protein phosphatase 2, regulatory subunit B', epsilon Protein phosphatase 2, regulatory subunit A, alpha	0.25 0.26 0.31 0.37	1.94×10^{-2} 4.25×10^{-2} 2.85×10^{-2} 3.87×10^{-2}	Positive regulator of Wnt/β-catenin signaling [52], Inhibition leads to spatial memory impairment [53]
	Csnk1a1 *Csnk1g2*	ENSMUST00000165123 ENSMUST00000085435	Casein kinase 1, alpha 1 Casein kinase 1, gamma 2	0.26 0.33	3.90×10^{-2} 4.73×10^{-2}	Negative regulators of SMAD3 and TGF-β signaling [54]
	Ctnnb1	ENSMUST00000007130	Catenin (cadherin associated protein), beta 1	0.37	1.38×10^{-2}	Positive regulator of Wnt/β-catenin signaling, neuronal survival and synaptic plasticity, negative regulator of Aβ production [55]
	Gsk3b	ENSMUST00000023507	Glycogen synthase kinase 3 beta	0.52	3.62×10^{-3}	Regulator of Wnt/β-catenin signaling, loss leads to synaptic and social defects in mice [56]
	Dvl3	ENSMUST00000003318	Dishevelled segment polarity protein 3	0.72	2.23×10^{-2}	Positive regulator of Wnt/β-catenin signaling, downregulated in AD brain [55]
	Fzd1	ENSMUST00000054294	Frizzled class receptor 1	0.73	3.81×10^{-3}	Positive regulator of Wnt/β-catenin signaling, loss leads to impairment of neuronal differentiation [57]

References

1. Stojakovic, A.; Trushin, S.; Sheu, A.; Khalili, L.; Chang, S.Y.; Li, X.; Christensen, T.; Salisbury, J.L.; Geroux, R.E.; Gateno, B.; et al. Partial inhibition of mitochondrial complex I ameliorates Alzheimer's disease pathology and cognition in APP/PS1 female mice. *Commun Biol* **2021**, *4*, 61, doi:10.1038/s42003-020-01584-y.
2. Schiff, M.; Haberberger, B.; Xia, C.; Mohsen, A.W.; Goetzman, E.S.; Wang, Y.; Uppala, R.; Zhang, Y.; Karunanidhi, A.; Prabhu, D.; et al. Complex I assembly function and fatty acid oxidation enzyme activity of ACAD9 both contribute to disease severity in ACAD9 deficiency. *Hum Mol Genet* **2015**, *24*, 3238-3247, doi:10.1093/hmg/ddv074.

3. Kumar, R.; Corbett, M.A.; Smith, N.J.C.; Hock, D.H.; Kikhtyak, Z.; Semcesen, L.N.; Morimoto, A.; Lee, S.; Stroud, D.A.; Gleeson, J.G.; et al. Oligonucleotide correction of an intronic TIMMDC1 variant in cells of patients with severe neurodegenerative disorder. *NPJ Genom Med* **2022**, *7*, 9, doi:10.1038/s41525-021-00277-7.
4. Fukui, H.; Diaz, F.; Garcia, S.; Moraes, C.T. Cytochrome c oxidase deficiency in neurons decreases both oxidative stress and amyloid formation in a mouse model of Alzheimer's disease. *Proc Natl Acad Sci U S A* **2007**, *104*, 14163-14168, doi:10.1073/pnas.0705738104.
5. Okazaki, M.; Ishibashi, Y.; Asoh, S.; Ohta, S. Overexpressed mitochondrial hinge protein, a cytochrome c-binding protein, accelerates apoptosis by enhancing the release of cytochrome c from mitochondria. *Biochem Biophys Res Commun* **1998**, *243*, 131-136, doi:10.1006/bbrc.1997.7979.
6. Kim, S.H.; Vlkolinsky, R.; Cairns, N.; Lubec, G. Decreased levels of complex III core protein 1 and complex V beta chain in brains from patients with Alzheimer's disease and Down syndrome. *Cell Mol Life Sci* **2000**, *57*, 1810-1816, doi:10.1007/pl00000661.
7. Pomytkin, I.; Pinelis, V. Brain Insulin Resistance: Focus on Insulin Receptor-Mitochondria Interactions. *Life (Basel)* **2021**, *11*, doi:10.3390/life11030262.
8. Richman, T.R.; Spahr, H.; Ermer, J.A.; Davies, S.M.; Viola, H.M.; Bates, K.A.; Papadimitriou, J.; Hool, L.C.; Rodger, J.; Larsson, N.G.; et al. Loss of the RNA-binding protein TACO1 causes late-onset mitochondrial dysfunction in mice. *Nat Commun* **2016**, *7*, 11884, doi:10.1038/ncomms11884.
9. Mangialasche, F.; Baglioni, M.; Cecchetti, R.; Kivipelto, M.; Ruggiero, C.; Piobbico, D.; Kussmaul, L.; Monastero, R.; Brancorsini, S.; Mecocci, P. Lymphocytic mitochondrial aconitase activity is reduced in Alzheimer's disease and mild cognitive impairment. *J Alzheimers Dis* **2015**, *44*, 649-660, doi:10.3233/JAD-142052.
10. Seim, G.L.; Britt, E.C.; John, S.V.; Yeo, F.J.; Johnson, A.R.; Eisenstein, R.S.; Pagliarini, D.J.; Fan, J. Two-stage metabolic remodelling in macrophages in response to lipopolysaccharide and interferon-gamma stimulation. *Nat Metab* **2019**, *1*, 731-742, doi:10.1038/s42255-019-0083-2.
11. Fisar, Z.; Hroudova, J.; Hansikova, H.; Spacilova, J.; Lelkova, P.; Wenchich, L.; Jirak, R.; Zverova, M.; Zeman, J.; Martasek, P.; et al. Mitochondrial Respiration in the Platelets of Patients with Alzheimer's Disease. *Curr Alzheimer Res* **2016**, *13*, 930-941, doi:10.2174/1567205013666160314150856.
12. Nakai, N.; Obayashi, M.; Nagasaki, M.; Sato, Y.; Fujitsuka, N.; Yoshimura, A.; Miyazaki, Y.; Sugiyama, S.; Shimomura, Y. The abundance of mRNAs for pyruvate dehydrogenase kinase isoenzymes in brain regions of young and aged rats. *Life Sci* **2000**, *68*, 497-503, doi:10.1016/s0024-3205(00)00947-4.
13. Izumi, K.; Brett, M.; Nishi, E.; Drunat, S.; Tan, E.S.; Fujiki, K.; Lebon, S.; Cham, B.; Masuda, K.; Arakawa, M.; et al. ARCN1 Mutations Cause a Recognizable Craniofacial Syndrome Due to COPI-Mediated Transport Defects. *Am J Hum Genet* **2016**, *99*, 451-459, doi:10.1016/j.ajhg.2016.06.011.
14. LaMonte, B.H.; Wallace, K.E.; Holloway, B.A.; Shelly, S.S.; Ascano, J.; Tokito, M.; Van Winkle, T.; Howland, D.S.; Holzbaur, E.L. Disruption of dynein/dynactin inhibits axonal transport in motor neurons causing late-onset progressive degeneration. *Neuron* **2002**, *34*, 715-727, doi:10.1016/s0896-6273(02)00696-7.
15. Cuddy, L.K.; Wani, W.Y.; Morella, M.L.; Pitcairn, C.; Tsutsumi, K.; Fredriksen, K.; Justman, C.J.; Grammatopoulos, T.N.; Belur, N.R.; Zunke, F.; et al. Stress-Induced Cellular Clearance Is Mediated by the SNARE Protein ykt6 and Disrupted by alpha-Synuclein. *Neuron* **2019**, *104*, 869-884 e811, doi:10.1016/j.neuron.2019.09.001.
16. Trychta, K.A.; Back, S.; Henderson, M.J.; Harvey, B.K. KDEL Receptors Are Differentially Regulated to Maintain the ER Proteome under Calcium Deficiency. *Cell Rep* **2018**, *25*, 1829-1840 e1826, doi:10.1016/j.celrep.2018.10.055.
17. Akama, T.O.; Nakagawa, H.; Wong, N.K.; Sutton-Smith, M.; Dell, A.; Morris, H.R.; Nakayama, J.; Nishimura, S.; Pai, A.; Moremen, K.W.; et al. Essential and mutually compensatory roles of {alpha}-mannosidase II and {alpha}-mannosidase IIx in N-glycan processing in vivo in mice. *Proc Natl Acad Sci U S A* **2006**, *103*, 8983-8988, doi:10.1073/pnas.0603248103.

18. Jain Goyal, M.; Zhao, X.; Bozhinova, M.; Andrade-Lopez, K.; de Heus, C.; Schulze-Dramac, S.; Muller-McNicoll, M.; Klumperman, J.; Bethune, J. A paralog-specific role of COPI vesicles in the neuronal differentiation of mouse pluripotent cells. *Life Sci Alliance* **2020**, *3*, doi:10.26508/lsa.202000714.
19. Sidjanin, D.J.; Park, A.K.; Ronchetti, A.; Martins, J.; Jackson, W.T. TBC1D20 mediates autophagy as a key regulator of autophagosome maturation. *Autophagy* **2016**, *12*, 1759-1775, doi:10.1080/15548627.2016.1199300.
20. Climer, L.K.; Dobretsov, M.; Lupashin, V. Defects in the COG complex and COG-related trafficking regulators affect neuronal Golgi function. *Front Neurosci* **2015**, *9*, 405, doi:10.3389/fnins.2015.00405.
21. Jepson, J.E.C.; Praschberger, R.; Krishnakumar, S.S. Mechanisms of Neurological Dysfunction in GOSR2 Progressive Myoclonus Epilepsy, a Golgi SNAREopathy. *Neuroscience* **2019**, *420*, 41-49, doi:10.1016/j.neuroscience.2019.03.057.
22. Zhao, J.; Wang, Y.; Xu, H.; Fu, Y.; Qian, T.; Bo, D.; Lu, Y.X.; Xiong, Y.; Wan, J.; Zhang, X.; et al. Dync1h1 Mutation Causes Proprioceptive Sensory Neuron Loss and Impaired Retrograde Axonal Transport of Dorsal Root Ganglion Neurons. *CNS Neurosci Ther* **2016**, *22*, 593-601, doi:10.1111/cns.12552.
23. Schrotter, A.; Oberhaus, A.; Kolbe, K.; Seger, S.; Mastalski, T.; El Magraoui, F.; Hoffmann-Posorske, E.; Bohnert, M.; Deckert, J.; Braun, C.; et al. LMD proteomics provides evidence for hippocampus field-specific motor protein abundance changes with relevance to Alzheimer's disease. *Biochim Biophys Acta Proteins Proteom* **2017**, *1865*, 703-714, doi:10.1016/j.bbapap.2017.03.013.
24. Berchtold, N.C.; Coleman, P.D.; Cribbs, D.H.; Rogers, J.; Gillen, D.L.; Cotman, C.W. Synaptic genes are extensively downregulated across multiple brain regions in normal human aging and Alzheimer's disease. *Neurobiol Aging* **2013**, *34*, 1653-1661, doi:10.1016/j.neurobiolaging.2012.11.024.
25. Wang, B.; Joo, J.H.; Mount, R.; Teubner, B.J.W.; Krenzer, A.; Ward, A.L.; Ichhaporia, V.P.; Adams, E.J.; Khoriaty, R.; Peters, S.T.; et al. The COPII cargo adapter SEC24C is essential for neuronal homeostasis. *J Clin Invest* **2018**, *128*, 3319-3332, doi:10.1172/JCI98194.
26. Li, S.; Yan, R.; Xu, J.; Zhao, S.; Ma, X.; Sun, Q.; Zhang, M.; Li, Y.; Liu, J.G.; Chen, L.; et al. A new type of ERGIC-ERES membrane contact mediated by TMED9 and SEC12 is required for autophagosome biogenesis. *Cell Res* **2022**, *32*, 119-138, doi:10.1038/s41422-021-00563-0.
27. Naydenov, N.G.; Harris, G.; Brown, B.; Schaefer, K.L.; Das, S.K.; Fisher, P.B.; Ivanov, A.I. Loss of soluble N-ethylmaleimide-sensitive factor attachment protein alpha (alphaSNAP) induces epithelial cell apoptosis via down-regulation of Bcl-2 expression and disruption of the Golgi. *J Biol Chem* **2012**, *287*, 5928-5941, doi:10.1074/jbc.M111.278358.
28. Akila Parvathy Dharshini, S.; Taguchi, Y.H.; Michael Gromiha, M. Exploring the selective vulnerability in Alzheimer disease using tissue specific variant analysis. *Genomics* **2019**, *111*, 936-949, doi:10.1016/j.ygeno.2018.05.024.
29. Liang, Z.S.; Cimino, I.; Yalcin, B.; Raghupathy, N.; Vancollie, V.E.; Ibarra-Soria, X.; Firth, H.V.; Rimmington, D.; Farooqi, I.S.; Lelliott, C.J.; et al. Trappc9 deficiency causes parent-of-origin dependent microcephaly and obesity. *PLoS Genet* **2020**, *16*, e1008916, doi:10.1371/journal.pgen.1008916.
30. Helferich, A.M.; Brockmann, S.J.; Reinders, J.; Deshpande, D.; Holzmann, K.; Brenner, D.; Andersen, P.M.; Petri, S.; Thal, D.R.; Michaelis, J.; et al. Dysregulation of a novel miR-1825/TBCB/TUBA4A pathway in sporadic and familial ALS. *Cell Mol Life Sci* **2018**, *75*, 4301-4319, doi:10.1007/s00018-018-2873-1.
31. Ong, Y.S.; Tran, T.H.; Gounko, N.V.; Hong, W. TMEM115 is an integral membrane protein of the Golgi complex involved in retrograde transport. *J Cell Sci* **2014**, *127*, 2825-2839, doi:10.1242/jcs.136754.
32. Chornyy, S.; Parnis, A.; Shmoish, M.; Cassel, D. High abundance of ArfGAP1 found in the mossy fibers in hilus of the dentate gyrus region of the mouse brain. *PLoS One* **2017**, *12*, e0189659, doi:10.1371/journal.pone.0189659.
33. Mkhikian, H.; Grigorian, A.; Li, C.F.; Chen, H.L.; Newton, B.; Zhou, R.W.; Beeton, C.; Torossian, S.; Tatarian, G.G.; Lee, S.U.; et al. Genetics and the environment converge to dysregulate N-glycosylation in multiple sclerosis. *Nat Commun* **2011**, *2*, 334, doi:10.1038/ncomms1333.

34. Wang, X.; Shi, N.; Hui, M.; Jin, H.; Gao, S.; Zhou, Q.; Zhang, L.; Yan, M.; Shen, H. The Impact of beta-1,4-Galactosyltransferase V on Microglial Function. *Front Cell Neurosci* **2021**, *15*, 723308, doi:10.3389/fncel.2021.723308.
35. Sapperstein, S.K.; Walter, D.M.; Grosvenor, A.R.; Heuser, J.E.; Waters, M.G. p115 is a general vesicular transport factor related to the yeast endoplasmic reticulum to Golgi transport factor Uso1p. *Proc Natl Acad Sci U S A* **1995**, *92*, 522-526, doi:10.1073/pnas.92.2.522.
36. Lin, C.Y.; Lai, H.L.; Chen, H.M.; Siew, J.J.; Hsiao, C.T.; Chang, H.C.; Liao, K.S.; Tsai, S.C.; Wu, C.Y.; Kitajima, K.; et al. Functional roles of ST8SIA3-mediated sialylation of striatal dopamine D2 and adenosine A2A receptors. *Transl Psychiatry* **2019**, *9*, 209, doi:10.1038/s41398-019-0529-z.
37. Herring, B.E.; Shi, Y.; Suh, Y.H.; Zheng, C.Y.; Blankenship, S.M.; Roche, K.W.; Nicoll, R.A. Cornichon proteins determine the subunit composition of synaptic AMPA receptors. *Neuron* **2013**, *77*, 1083-1096, doi:10.1016/j.neuron.2013.01.017.
38. Sannerud, R.; Declerck, I.; Peric, A.; Raemaekers, T.; Menendez, G.; Zhou, L.; Veerle, B.; Coen, K.; Munck, S.; De Strooper, B.; et al. ADP ribosylation factor 6 (ARF6) controls amyloid precursor protein (APP) processing by mediating the endosomal sorting of BACE1. *Proc Natl Acad Sci U S A* **2011**, *108*, E559-568, doi:10.1073/pnas.1100745108.
39. Ziembik, M.A.; Bender, T.P.; Larner, J.M.; Brautigan, D.L. Functions of protein phosphatase-6 in NF-kappaB signaling and in lymphocytes. *Biochem Soc Trans* **2017**, *45*, 693-701, doi:10.1042/BST20160169.
40. Noel, J.; Ralph, G.S.; Pickard, L.; Williams, J.; Molnar, E.; Uney, J.B.; Collingridge, G.L.; Henley, J.M. Surface expression of AMPA receptors in hippocampal neurons is regulated by an NSF-dependent mechanism. *Neuron* **1999**, *23*, 365-376, doi:10.1016/s0896-6273(00)80786-2.
41. Efimova, N.; Korobova, F.; Stankewich, M.C.; Moberly, A.H.; Stolz, D.B.; Wang, J.; Kashina, A.; Ma, M.; Svitkina, T. betaIII Spectrin Is Necessary for Formation of the Constricted Neck of Dendritic Spines and Regulation of Synaptic Activity in Neurons. *J Neurosci* **2017**, *37*, 6442-6459, doi:10.1523/JNEUROSCI.3520-16.2017.
42. Gao, Z.H.; Seeling, J.M.; Hill, V.; Yochum, A.; Virshup, D.M. Casein kinase I phosphorylates and destabilizes the beta-catenin degradation complex. *Proc Natl Acad Sci U S A* **2002**, *99*, 1182-1187, doi:10.1073/pnas.032468199.
43. Bettencourt, C.; Foti, S.C.; Miki, Y.; Botia, J.; Chatterjee, A.; Warner, T.T.; Revesz, T.; Lashley, T.; Balazs, R.; Vire, E.; et al. White matter DNA methylation profiling reveals deregulation of HIP1, LMAN2, MOBP, and other loci in multiple system atrophy. *Acta Neuropathol* **2020**, *139*, 135-156, doi:10.1007/s00401-019-02074-0.
44. Mendoza-Ferreira, N.; Karakaya, M.; Cengiz, N.; Beijer, D.; Brigatti, K.W.; Gonzaga-Jauregui, C.; Fuhrmann, N.; Holker, I.; Thelen, M.P.; Zetzsche, S.; et al. De Novo and Inherited Variants in GBF1 are Associated with Axonal Neuropathy Caused by Golgi Fragmentation. *Am J Hum Genet* **2020**, *107*, 763-777, doi:10.1016/j.ajhg.2020.08.018.
45. Lu, X.; Zhang, D.; Shoji, H.; Duan, C.; Zhang, G.; Isaji, T.; Wang, Y.; Fukuda, T.; Gu, J. Deficiency of alpha1,6-fucosyltransferase promotes neuroinflammation by increasing the sensitivity of glial cells to inflammatory mediators. *Biochim Biophys Acta Gen Subj* **2019**, *1863*, 598-608, doi:10.1016/j.bbagen.2018.12.008.
46. Rehfeld, A.; Plass, M.; Krogh, A.; Friis-Hansen, L. Alterations in polyadenylation and its implications for endocrine disease. *Front Endocrinol (Lausanne)* **2013**, *4*, 53, doi:10.3389/fendo.2013.00053.
47. Alcott, C.E.; Yalamanchili, H.K.; Ji, P.; van der Heijden, M.E.; Saltzman, A.; Elrod, N.; Lin, A.; Leng, M.; Bhatt, B.; Hao, S.; et al. Partial loss of CFIm25 causes learning deficits and aberrant neuronal alternative polyadenylation. *Elife* **2020**, *9*, doi:10.7554/eLife.50895.
48. Gebhardt, A.; Habjan, M.; Benda, C.; Meiler, A.; Haas, D.A.; Hein, M.Y.; Mann, A.; Mann, M.; Habermann, B.; Pichlmair, A. mRNA export through an additional cap-binding complex consisting of NCBP1 and NCBP3. *Nat Commun* **2015**, *6*, 8192, doi:10.1038/ncomms9192.

49. Schonemann, L.; Kuhn, U.; Martin, G.; Schafer, P.; Gruber, A.R.; Keller, W.; Zavolan, M.; Wahle, E. Reconstitution of CPSF active in polyadenylation: recognition of the polyadenylation signal by WDR33. *Genes Dev* **2014**, *28*, 2381-2393, doi:10.1101/gad.250985.114.
50. Cappell, K.M.; Larson, B.; Sciaky, N.; Whitehurst, A.W. Symplekin specifies mitotic fidelity by supporting microtubule dynamics. *Mol Cell Biol* **2010**, *30*, 5135-5144, doi:10.1128/MCB.00758-10.
51. Rosenthal, S.B.; Wang, H.; Shi, D.; Liu, C.; Abagyan, R.; McEvoy, L.K.; Chen, C.H. Mapping the gene network landscape of Alzheimer's disease through integrating genomics and transcriptomics. *PLoS Comput Biol* **2022**, *18*, e1009903, doi:10.1371/journal.pcbi.1009903.
52. Bos, C.L.; Kodach, L.L.; van den Brink, G.R.; Diks, S.H.; van Santen, M.M.; Richel, D.J.; Peppelenbosch, M.P.; Hardwick, J.C. Effect of aspirin on the Wnt/beta-catenin pathway is mediated via protein phosphatase 2A. *Oncogene* **2006**, *25*, 6447-6456, doi:10.1038/sj.onc.1209658.
53. Sun, L.; Liu, S.Y.; Zhou, X.W.; Wang, X.C.; Liu, R.; Wang, Q.; Wang, J.Z. Inhibition of protein phosphatase 2A- and protein phosphatase 1-induced tau hyperphosphorylation and impairment of spatial memory retention in rats. *Neuroscience* **2003**, *118*, 1175-1182, doi:10.1016/s0306-4522(02)00697-8.
54. Guo, X.; Waddell, D.S.; Wang, W.; Wang, Z.; Liberati, N.T.; Yong, S.; Liu, X.; Wang, X.F. Ligand-dependent ubiquitination of Smad3 is regulated by casein kinase 1 gamma 2, an inhibitor of TGF-beta signaling. *Oncogene* **2008**, *27*, 7235-7247, doi:10.1038/onc.2008.337.
55. Jia, L.; Pina-Crespo, J.; Li, Y. Restoring Wnt/beta-catenin signaling is a promising therapeutic strategy for Alzheimer's disease. *Mol Brain* **2019**, *12*, 104, doi:10.1186/s13041-019-0525-5.
56. Wang, M.; Liu, X.; Hou, Y.; Zhang, H.; Kang, J.; Wang, F.; Zhao, Y.; Chen, J.; Liu, X.; Wang, Y.; et al. Decrease of GSK-3beta Activity in the Anterior Cingulate Cortex of Shank3b (-/-) Mice Contributes to Synaptic and Social Deficiency. *Front Cell Neurosci* **2019**, *13*, 447, doi:10.3389/fncel.2019.00447.
57. Mardones, M.D.; Andaur, G.A.; Varas-Godoy, M.; Henriquez, J.F.; Salech, F.; Behrens, M.I.; Couve, A.; Inestrosa, N.C.; Varela-Nallar, L. Frizzled-1 receptor regulates adult hippocampal neurogenesis. *Mol Brain* **2016**, *9*, 29, doi:10.1186/s13041-016-0209-3.
58. Goldberg, J.; Currais, A.; Prior, M.; Fischer, W.; Chiruta, C.; Ratliff, E.; Daugherty, D.; Dargusch, R.; Finley, K.; Esparza-Molto, P.B.; et al. The mitochondrial ATP synthase is a shared drug target for aging and dementia. *Aging Cell* **2018**, *17*, doi:10.1111/acel.12715.
59. Ferre, C.A.; Thouard, A.; Betourne, A.; Le Dorze, A.L.; Belenguer, P.; Miquel, M.C.; Peyrin, J.M.; Gonzalez-Dunia, D.; Szelechowski, M. HSPA9/Mortalin mediates axo-protection and modulates mitochondrial dynamics in neurons. *Sci Rep* **2021**, *11*, 17705, doi:10.1038/s41598-021-97162-1.
60. Silva, P.N.; Furuya, T.K.; Braga, I.L.; Rasmussen, L.T.; Labio, R.W.; Bertolucci, P.H.; Chen, E.S.; Turecki, G.; Mechawar, N.; Payao, S.L.; et al. Analysis of HSPA8 and HSPA9 mRNA expression and promoter methylation in the brain and blood of Alzheimer's disease patients. *J Alzheimers Dis* **2014**, *38*, 165-170, doi:10.3233/JAD-130428.
61. Jian, F.; Chen, D.; Chen, L.; Yan, C.; Lu, B.; Zhu, Y.; Chen, S.; Shi, A.; Chan, D.C.; Song, Z. Sam50 Regulates PINK1-Parkin-Mediated Mitophagy by Controlling PINK1 Stability and Mitochondrial Morphology. *Cell Rep* **2018**, *23*, 2989-3005, doi:10.1016/j.celrep.2018.05.015.
